# Supplementary material for: A Likelihood Approach for Real-Time Calibration of Stochastic Compartmental Epidemic Models
Source: PLoS Comput Biol. 2017 Jan 17;13(1):e1005257. doi: 10.1371/journal.pcbi.1005257 (PMC5240920; doi:10.1371/journal.pcbi.1005257)
Supplement: S1 File — (TAR.GZ) [file pcbi.1005257.s014.tar.gz › HSPH_Online-SI-Revision/MSS10/n100-mild/n100-mild_NEW_table-4.pdf]

1	R0	{1.24125, 3.38982, 0.1}	{1.22618, 3.08238, 0.01}	{{1.22544, 3.07157, 0.}, {1.22544, 3.07157, 0.}}	{1.2795, 9.17807, 108.}	inf	1007.	877.433	{709.685, 1018.55}	14.8723	0.0730504	newly	2409.	2338.11	{1882., 2744.}	11.6775	0.0531354	Reff	1.16056	1.14805	{1.14513, 1.14951}	1.14194	ar	34926.	33726.2	{32270., 35338.}	4.3459
2	R0	{1.29554, 4.51878, 0.1}	{1.22502, 3.08664, 0.03}	{{1.22544, 3.07157, 0.}, {1.22544, 3.07157, 0.}}	{5.44316, 31.693, 124.}	inf	1718.	1157.57	{968.746, 1328.51}	32.6213	0.174192	newly	2989.	2939.12	{2486., 3352.}	9.08866	0.0405267	Reff	1.16829	1.11157	{1.10975, 1.11417}	4.85546	ar	42828.	33717.	{32271., 35118.}	21.2736
3	R0	{1.2443, 3.64292, 0.1}	{1.24045, 2.72485, 0.06}	{{1.22544, 2.5364, 0.}, {1.24751, 3.07157, 0.}}	{0.735677, 25.2015, 156.}	inf	1255.	980.607	{779.034, 1213.31}	22.7346	0.118359	newly	2853.	3046.44	{2434., 3597.}	14.497	0.0601821	Reff	1.15569	1.15552	{1.13868, 1.16528}	0.925392	ar	37087.	35326.6	{33601., 37076.}	5.09828
4	R0	{1.38465, 3.43331, 0.1}	{1.26819, 1.43018, 3.1}	{{1.2663, 1.35135, 3.}, {1.27121, 1.91565, 4.}}	{8.42102, 58.3439, 3002.}	inf	1365.	593.241	{123.222, 1027.12}	56.6349	0.461532	newly	3916.	4728.3	{1095., 8065.}	55.1522	0.247331	Reff	1.30897	1.20865	{1.20136, 1.21292}	7.68716	ar	49511.	37896.	{35841., 39580.}	23.4595
5	R0	{1.25409, 4.1068, 0.1}	{1.22895, 3.09472, 0.}	{{1.22544, 3.07157, 0.}, {1.25474, 3.26445, 0.}}	{2.01681, 24.6441, 100.}	inf	1456.	1112.08	{951.948, 1255.06}	23.6208	0.119775	newly	2839.	2922.69	{2453., 3305.}	9.37408	0.0400201	Reff	1.15606	1.13711	{1.13236, 1.15707}	1.71672	ar	38571.	34485.2	{32828., 35931.}	10.6364
6	R0	{1.28821, 4.2016, 0.1}	{1.22712, 3.07586, 0.}	{{1.22544, 3.07157, 0.}, {1.22544, 3.07157, 0.}}	{4.74202, 26.793, 100.}	inf	1742.	1274.04	{1103.65, 1474.82}	26.9209	0.139327	newly	3382.	3249.37	{2782., 3798.}	10.1789	0.0458149	Reff	1.16363	1.11419	{1.1102, 1.11568}	4.24862	ar	41468.	34162.9	{32471., 35960.}	17.6162
7	R0	{1.24369, 2.56007, 0.1}	{1.2533, 2.58584, 0.}	{{1.24751, 2.5364, 0.}, {1.24751, 2.5364, 0.}}	{0.801688, 2.78129, 100.}	inf	601.	630.361	{470.681, 800.611}	19.1661	0.0796934	newly	2262.	2260.86	{1631., 2933.}	17.107	0.0759012	Reff	1.20376	1.21266	{1.20497, 1.20921}	0.77001	ar	38603.	37278.7	{35046., 38385.}	6.05287
8	R0	{1.232, 3.53942, 0.1}	{1.22632, 3.07736, 0.}	{{1.22544, 3.07157, 0.}, {1.22544, 3.07157, 0.}}	{0.572104, 13.0547, 100.}	inf	1387.	1248.18	{1034.17, 1437.23}	12.2303	0.0588172	newly	3108.	3195.34	{2610., 3659.}	10.4858	0.0449352	Reff	1.11866	1.1152	{1.11199, 1.11772}	0.425765	ar	34555.	34074.4	{32517., 35204.}	2.77804
9	R0	{1.36262, 3.85631, 0.1}	{1.24751, 2.5364, 0.}	{{1.24751, 2.5364, 0.}, {1.24751, 2.5364, 0.}}	{8.44748, 34.2272, 100.}	inf	1592.	994.853	{703.611, 1211.16}	37.5092	0.211841	newly	3831.	3439.83	{2566., 4123.}	14.8831	0.0723098	Reff	1.27209	1.17208	{1.16915, 1.17537}	7.86187	ar	48410.	36686.8	{35315., 38163.}	24.2164
10	R0	{1.28702, 4.67968, 1.}	{1.20331, 2.96556, 0.97}	{{1.12171, 1.78492, 0.}, {1.22544, 3.57392, 4.}}	{6.50446, 36.629, 111.}	inf	1874.	1114.58	{597.77, 1407.88}	40.5238	0.244247	newly	3114.	2824.61	{2208., 3470.}	14.7672	0.0714966	Reff	1.13924	1.0742	{1.00747, 1.09531}	5.70936	ar	41092.	30767.	{21257., 34368.}	25.1266
11	R0	{1.35752, 3.47129, 0.1}	{1.24751, 2.5364, 0.}	{{1.24751, 2.5364, 0.}, {1.24751, 2.5364, 0.}}	{8.10353, 26.9319, 100.}	inf	974.	756.331	{530.432, 956.377}	23.8864	0.128521	newly	2552.	2700.02	{1870., 3501.}	19.1724	0.0822214	Reff	1.29461	1.19242	{1.18985, 1.19507}	7.89378	ar	44669.	36703.2	{35429., 38064.}	17.833
12	R0	{1.35929, 5.83647, 0.1}	{1.2259, 3.08573, 0.01}	{{1.22544, 3.07157, 0.}, {1.22544, 3.07157, 0.}}	{9.81294, 47.1302, 108.}	inf	2459.	1315.91	{1148.36, 1483.8}	46.4859	0.274112	newly	3330.	3279.43	{2869., 3673.}	7.63634	0.034204	Reff	1.20052	1.09673	{1.09417, 1.09837}	8.64574	ar	46715.	33866.2	{32558., 35257.}	27.5047
13	R0	{1.28019, 3.29908, 0.1}	{1.24751, 2.5364, 0.}	{{1.24751, 2.5364, 0.}, {1.24751, 2.5364, 0.}}	{2.55251, 23.1178, 100.}	inf	1185.	890.504	{638.43, 1124.8}	25.3085	0.13553	newly	3104.	3113.18	{2237., 3897.}	16.6591	0.0746406	Reff	1.21037	1.18315	{1.1802, 1.18615}	2.24898	ar	40941.	36833.9	{35290., 38320.}	10.0318
14	R0	{1.36661, 3.48318, 0.1}	{1.26061, 2.627, 0.}	{{1.24751, 2.5364, 0.}, {1.24751, 2.5364, 0.}}	{8.62735, 26.5205, 100.}	inf	1869.	1366.52	{1051.69, 1637.4}	28.7054	0.151912	newly	4569.	4456.52	{3528., 5218.}	11.7247	0.0534324	Reff	1.26507	1.17318	{1.15865, 1.16622}	8.09538	ar	48672.	38518.9	{36065., 39037.}	22.6565
15	R0	{1.35865, 3.84123, 0.1}	{1.24751, 2.5364, 0.}	{{1.24751, 2.5364, 0.}, {1.24751, 2.5364, 0.}}	{8.17996, 33.9689, 100.}	inf	1321.	948.001	{700.1, 1193.35}	28.3457	0.155691	newly	3065.	3315.67	{2410., 4175.}	19.0261	0.0810342	Reff	1.28034	1.18026	{1.1772, 1.18335}	7.81652	ar	48598.	36836.1	{35464., 38216.}	24.2024
16	R0	{1.22879, 3.98329, 0.1}	{1.21818, 2.98151, 0.28}	{{1.22544, 3.07157, 0.}, {1.22544, 3.07157, 0.}}	{0.863744, 25.1497, 366.}	inf	1186.	958.004	{771.229, 1146.17}	19.6125	0.102296	newly	2363.	2552.76	{2056., 2965.}	14.4037	0.0591887	Reff	1.12545	1.11848	{1.12228, 1.12686}	0.685348	ar	34405.	32382.8	{30493., 34627.}	6.30513
17	R0	{1.34925, 3.64524, 0.1}	{1.24751, 2.5364, 0.}	{{1.24751, 2.5364, 0.}, {1.24751, 2.5364, 0.}}	{7.54026, 30.4187, 100.}	inf	1082.	755.865	{525.405, 986.777}	30.2307	0.168041	newly	2774.	2700.93	{1889., 3440.}	18.3032	0.0841068	Reff	1.28476	1.19195	{1.18893, 1.19474}	7.22358	ar	46871.	36588.8	{35043., 38081.}	21.9372
18	R0	{1.33054, 2.25275, 0.1}	{1.3921, 1.9059, 0.01}	{{1.39162, 1.89883, 0.}, {1.39162, 1.89883, 0.}}	{4.62705, 15.7103, 108.}	inf	387.	393.411	{193.425, 590.035}	34.9292	0.170263	newly	1979.	3011.4	{1542., 4595.}	65.0328	0.220384	Reff	1.30961	1.37012	{1.36689, 1.37243}	4.6202	ar	44336.	50408.	{49232., 51454.}	13.6953
19	R0	{1.28394, 3.69859, 0.1}	{1.23094, 3.04655, 0.}	{{1.22544, 3.07157, 0.}, {1.25474, 3.26445, 0.}}	{4.12791, 17.6294, 100.}	inf	1262.	1097.78	{892.926, 1283.74}	14.418	0.0701882	newly	2837.	2941.14	{2421., 3388.}	10.8629	0.0459351	Reff	1.18844	1.1414	{1.13457, 1.16054}	3.95801	ar	39558.	34772.7	{32722., 36640.}	12.0969
20	R0	{1.2883, 2.26493, 0.1}	{1.51929, 3.91589, 0.1}	{{1.47608, 3.72349, 0.}, {1.53501, 4.02511, 0.}}	{18.1202, 73.2155, 186.}	inf	248.	508.658	{371.915, 646.298}	105.583	0.303491	newly	1268.	1457.82	{1065., 1784.}	22.194	0.0854349	Reff	1.27318	1.49746	{1.45275, 1.51475}	17.812	ar	40768.	59119.2	{55887., 61177.}	45.4405
21	R0	{1.34098, 2.78621, 0.1}	{1.25146, 2.52956, 0.09}	{{1.24751, 2.5364, 0.}, {1.24751, 2.5364, 0.}}	{6.97031, 9.21146, 182.}	inf	675.	592.473	{441.752, 756.345}	18.102	0.0898181	newly	2390.	2213.13	{1606., 2777.}	16.746	0.0791537	Reff	1.30397	1.21794	{1.21204, 1.21612}	6.88653	ar	44605.	37451.4	{35808., 38225.}	16.8352
22	R0	{1.33783, 5.95911, 0.1}	{1.21436, 3.47345, 0.8}	{{1.21159, 3.07157, 0.}, {1.22544, 3.57392, 1.}}	{9.22893, 41.712, 740.}	inf	2154.	1216.12	{1020.91, 1374.16}	43.5414	0.251544	newly	2912.	2662.75	{2262., 3030.}	11.0264	0.0516446	Reff	1.18802	1.08977	{1.08537, 1.10141}	8.26996	ar	45418.	32539.4	{31130., 33730.}	28.3557
23	R0	{1.33359, 3.3069, 0.1}	{1.24808, 2.64903, 0.}	{{1.24751, 2.5364, 0.}, {1.25474, 3.26445, 0.}}	{6.41178, 19.8937, 100.}	inf	965.	702.228	{464.572, 910.713}	27.688	0.154873	newly	2751.	2389.7	{1711., 3217.}	20.8382	0.104518	Reff	1.27359	1.19521	{1.19229, 1.19921}	6.15435	ar	46245.	36624.6	{34873., 38312.}	20.803
24	R0	{1.30588, 2.56123, 0.1}	{1.24501, 2.50411, 0.19}	{{1.24751, 2.5364, 0.}, {1.24751, 2.5364, 0.}}	{4.66151, 2.23021, 278.}	inf	683.	596.426	{388.248, 800.282}	20.0733	0.103672	newly	2554.	2221.7	{1465., 2908.}	19.7956	0.102208	Reff	1.26499	1.2071	{1.20646, 1.21177}	4.57639	ar	42383.	36715.3	{35093., 38403.}	13.3727
25	R0	{1.29813, 4.51849, 0.1}	{1.22447, 3.10674, 0.07}	{{1.22544, 3.07157, 0.}, {1.22544, 3.07157, 0.}}	{5.67447, 31.2439, 156.}	inf	1351.	963.422	{782.044, 1130.2}	28.6882	0.150733	newly	2397.	2527.44	{1991., 2974.}	12.8294	0.05361	Reff	1.19921	1.13591	{1.13397, 1.13865}	5.27871	ar	43371.	33678.4	{32029., 35164.}	22.3481
26	R0	{1.33231, 3.99953, 0.1}	{1.23363, 2.40889, 0.73}	{{1.19264, 2.01465, 0.}, {1.24751, 2.5364, 3.}}	{7.40638, 39.7708, 782.}	inf	1549.	951.649	{696.825, 1192.42}	38.5637	0.221063	newly	3228.	3367.78	{2586., 4115.}	15.334	0.0647515	Reff	1.2303	1.14652	{1.11019, 1.16106}	6.80925	ar	44733.	34513.9	{29308., 37164.}	22.8447
27	R0	{1.30555, 4.61445, 0.1}	{1.2269, 3.08122, 0.}	{{1.22544, 3.07157, 0.}, {1.22544, 3.07157, 0.}}	{6.02412, 33.2268, 100.}	inf	1634.	1025.44	{852.469, 1179.24}	37.2438	0.205971	newly	2878.	2717.15	{2296., 3196.}	10.9357	0.0504324	Reff	1.20568	1.14051	{1.13724, 1.14148}	5.40483	ar	42624.	34208.7	{32754., 35699.}	19.7431
28	R0	{1.26819, 5.34969, 0.1}	{1.2253, 3.0766, 0.01}	{{1.22544, 3.07157, 0.}, {1.22544, 3.07157, 0.}}	{3.3821, 42.4902, 108.}	inf	1779.	987.504	{806.689, 1151.58}	44.4911	0.260419	newly	2612.	2568.14	{2132., 3049.}	11.4824	0.0518955	Reff	1.1566	1.12718	{1.12524, 1.12945}	2.54357	ar	38946.	33631.7	{32270., 35041.}	13.6453
29	R0	{1.19925, 3.00394, 0.1}	{1.16193, 2.33446, 1.97}	{{1.06836, 1.78492, 0.}, {1.22544, 3.07157, 4.}}	{5.14926, 24.2162, 1956.}	inf	770.	633.715	{333.512, 951.901}	29.2318	0.163798	newly	2181.	2156.33	{1469., 2715.}	19.9033	0.0945073	Reff	1.12966	1.09598	{1.00999, 1.15493}	4.9515	ar	31727.	26057.6	{16024., 34766.}	24.7268
30	R0	{1.35749, 3.75483, 0.1}	{1.24751, 2.5364, 0.}	{{1.24751, 2.5364, 0.}, {1.24751, 2.5364, 0.}}	{8.1015, 32.4496, 100.}	inf	1440.	889.507	{653.844, 1114.59}	38.2287	0.216529	newly	3590.	3147.4	{2288., 3914.}	16.3928	0.0810034	Reff	1.28281	1.18575	{1.18293, 1.18865}	7.56615	ar	48244.	36965.	{35683., 38362.}	23.3791
31	R0	{1.27131, 4.2161, 0.1}	{1.22983, 3.1005, 0.}	{{1.22544, 3.07157, 0.}, {1.25474, 3.26445, 0.}}	{3.2626, 26.4604, 100.}	inf	1285.	972.407	{790.673, 1125.51}	24.3263	0.125315	newly	2456.	2598.86	{2162., 3032.}	12.408	0.0520216	Reff	1.18819	1.15327	{1.14744, 1.17525}	2.93927	ar	38299.	34740.4	{32795., 37041.}	9.35074
32	R0	{1.31827, 6.18119, 0.1}	{1.21346, 3.45556, 0.83}	{{1.21159, 3.07157, 0.}, {1.22544, 3.57392, 1.}}	{7.95028, 44.0956, 770.}	inf	2473.	1304.99	{1111.99, 1470.12}	47.2305	0.280565	newly	3092.	2848.57	{2485., 3139.}	9.54431	0.044327	Reff	1.16063	1.08253	{1.07861, 1.09542}	6.72923	ar	45262.	32658.3	{31230., 34078.}	27.8461
33	R0	{1.20398, 1.35167, 0.1}	{1.42176, 2.10281, 0.08}	{{1.39162, 1.89883, 0.}, {1.43965, 2.60601, 0.}}	{18.0884, 55.571, 164.}	inf	104.	231.63	{74.4285, 383.379}	136.114	0.363211	newly	1068.	1647.58	{542., 3041.}	77.691	0.272602	Reff	1.19578	1.41023	{1.37838, 1.43117}	17.9342	ar	33329.	52307.5	{49546., 55566.}	56.9428
34	R0	{1.32776, 3.146, 0.1}	{1.25179, 2.5664, 0.}	{{1.24751, 2.5364, 0.}, {1.24751, 2.5364, 0.}}	{6.12399, 19.5552, 100.}	inf	1228.	1084.72	{811.336, 1318.96}	16.3928	0.0801536	newly	3687.	3730.49	{2802., 4437.}	12.9897	0.0569091	Reff	1.25318	1.18324	{1.17631, 1.18264}	5.96211	ar	44872.	37539.8	{35895., 38317.}	17.3636
35	R0	{1.279, 1.11336, 0.1}	{1.45035, 1.34282, 0.96}	{{1.42206, 1.31573, 1.}, {1.42206, 1.31573, 1.}}	{13.3973, 20.6093, 868.}	inf	39.	123.659	{49.8908, 188.83}	221.152	0.47123	newly	625.	2515.	{809., 3760.}	305.168	0.544185	Reff	1.27363	1.44301	{1.41407, 1.41615}	13.2989	ar	41033.	54040.8	{51990., 53728.}	31.7009
36	R0	{1.20029, 1.94717, 0.1}	{1.39307, 1.92005, 0.03}	{{1.39162, 1.89883, 0.}, {1.39162, 1.89883, 0.}}	{16.0607, 3.42316, 124.}	inf	272.	419.471	{151.372, 639.519}	71.6027	0.244166	newly	1470.	3163.14	{1215., 4849.}	124.691	0.337386	Reff	1.18231	1.37014	{1.36566, 1.37277}	15.8868	ar	33234.	50378.6	{49003., 51459.}	51.5874
37	R0	{1.33216, 3.69685, 0.1}	{1.61068, 7.63397, 3.48}	{{1.19264, 2.01465, 3.}, {2.10451, 14.2406, 4.}}	{32.4315, 155.892, 3380.}	inf	1487.	2984.35	{512.525, 5691.43}	156.491	0.447161	newly	3432.	3191.22	{2286., 3700.}	12.2261	0.0633301	Reff	1.23359	1.4562	{1.11094, 1.86262}	28.7692	ar	45235.	53499.5	{27320., 82476.}	57.0717
38	R0	{1.25434, 2.04314, 0.1}	{1.26731, 1.72745, 2.15}	{{1.19264, 1.35135, 0.}, {1.39162, 2.01465, 3.}}	{4.80109, 18.3483, 2108.}	inf	320.	245.44	{47.3076, 423.463}	42.6096	0.316566	newly	1505.	1913.26	{346., 3747.}	81.5468	0.369104	Reff	1.23694	1.25066	{1.17634, 1.373}	4.80064	ar	38662.	37764.9	{28688., 50029.}	17.1016
39	R0	{1.27466, 3.0741, 0.1}	{1.24587, 2.52075, 0.09}	{{1.24751, 2.5364, 0.}, {1.24751, 2.5364, 0.}}	{2.25889, 18.0003, 184.}	inf	1446.	1169.32	{912.034, 1434.53}	19.8837	0.102029	newly	3912.	4014.36	{3110., 4810.}	14.3722	0.0623464	Reff	1.18848	1.16508	{1.16304, 1.16977}	1.96896	ar	40181.	36892.3	{35380., 38445.}	8.18476
40	R0	{1.33045, 2.68154, 0.1}	{1.42373, 2.3708, 0.67}	{{1.39162, 1.89883, 0.}, {1.43965, 2.60601, 1.}}	{7.01126, 11.9219, 638.}	inf	888.	849.165	{464.865, 1182.36}	23.1032	0.117825	newly	3300.	4443.65	{2805., 5968.}	42.4821	0.154012	Reff	1.28496	1.37558	{1.34566, 1.39229}	7.05205	ar	45951.	52738.9	{49611., 54861.}	14.772
41	R0	{1.37003, 2.83984, 0.1}	{1.35464, 3.11057, 0.03}	{{1.24751, 2.5364, 0.}, {1.53501, 4.02511, 0.}}	{9.42876, 22.1625, 128.}	inf	521.	566.291	{337.473, 840.33}	29.1399	0.123067	newly	2106.	1819.75	{1181., 2413.}	19.7146	0.101658	Reff	1.34277	1.32689	{1.22246, 1.50176}	9.29045	ar	48926.	46005.5	{36308., 60309.}	22.504
42	R0	{1.3003, 2.94885, 0.1}	{2.90125, 18.6058, 4.}	{{2.93183, 18.9272, 4.}, {2.93183, 18.9272, 4.}}	{123.122, 530.95, 3900.}	inf	940.	5186.42	{5073.12, 5393.85}	451.746	0.740906	newly	2995.	2679.63	{2586., 2762.}	10.5299	0.0485203	Reff	1.24816	2.66137	{2.68295, 2.69081}	113.224	ar	44237.	93235.2	{93324., 93683.}	110.763
43	R0	{1.33756, 2.92653, 0.1}	{1.28419, 2.4625, 0.18}	{{1.24751, 1.89883, 0.}, {1.43965, 2.60601, 1.}}	{6.55865, 15.8559, 254.}	inf	626.	434.958	{257.725, 577.878}	31.818	0.182312	newly	1978.	1881.6	{1194., 2393.}	26.5814	0.119889	Reff	1.31066	1.26082	{1.22311, 1.41104}	6.46767	ar	47342.	40479.6	{35981., 53581.}	19.1301
44	R0	{1.35801, 2.92276, 0.1}	{1.65592, 4.85944, 0.16}	{{1.53501, 4.02511, 0.}, {1.74592, 5.58477, 0.}}	{21.9375, 66.2619, 240.}	inf	645.	1099.63	{767.359, 1354.98}	70.5414	0.213327	newly	2455.	2484.28	{2126., 2782.}	8.7446	0.0379725	Reff	1.32668	1.60932	{1.49323, 1.69707}	21.3044	ar	47940.	65499.6	{59898., 71505.}	36.6283
45	R0	{1.31342, 4.02649, 0.1}	{1.2318, 3.07617, 0.03}	{{1.22544, 3.07157, 0.}, {1.25474, 3.26445, 0.}}	{6.21436, 23.6017, 128.}	inf	1370.	983.341	{778.311, 1202.66}	28.4994	0.15154	newly	2863.	2649.75	{2078., 3195.}	14.0045	0.0658089	Reff	1.22662	1.15515	{1.14686, 1.17474}	5.82654	ar	42995.	35067.1	{33130., 37534.}	18.4392
46	R0	{1.37919, 3.75889, 0.1}	{2.81635, 19.898, 4.}	{{2.81635, 19.898, 4.}, {2.81635, 19.898, 4.}}	{104.203, 429.358, 3900.}	inf	1025.	5017.76	{4888.43, 5147.28}	389.538	0.689696	newly	2667.	2351.38	{2276., 2419.}	11.8343	0.0548312	Reff	1.32725	2.59783	{2.59378, 2.60127}	95.7305	ar	49157.	92681.3	{92518., 92817.}	88.5413
47	R0	{1.37458, 3.56117, 0.1}	{1.30217, 2.05432, 2.36}	{{1.24751, 1.89883, 0.}, {1.39162, 2.5364, 4.}}	{5.97431, 42.3132, 2338.}	inf	1179.	852.668	{489.465, 1143.24}	29.7826	0.169099	newly	3193.	4543.27	{2830., 6886.}	47.7128	0.155953	Reff	1.31164	1.24676	{1.19333, 1.33337}	5.77388	ar	48478.	42126.1	{37349., 50570.}	15.248
48	R0	{1.33393, 3.78296, 0.1}	{1.25022, 3.28109, 0.}	{{1.22544, 3.07157, 0.}, {1.25474, 3.26445, 0.}}	{6.27519, 14.2285, 100.}	inf	1368.	1141.21	{948.323, 1321.72}	17.8532	0.0879706	newly	3101.	2891.81	{2505., 3279.}	10.8388	0.0500872	Reff	1.2473	1.17186	{1.14948, 1.17907}	6.04883	ar	44885.	37285.	{34392., 39516.}	16.9322
49	R0	{1.24918, 2.39046, 0.1}	{1.26665, 1.35143, 3.}	{{1.2663, 1.35135, 3.}, {1.2663, 1.35135, 3.}}	{1.39887, 43.4657, 2900.}	inf	662.	515.154	{98.3703, 950.727}	42.2017	0.284835	newly	2523.	4629.25	{954., 8008.}	105.033	0.32545	Reff	1.19869	1.21732	{1.21129, 1.22225}	1.554	ar	35969.	38001.2	{36084., 39865.}	5.91593
50	R0	{1.28281, 2.3032, 0.1}	{1.38912, 1.88788, 0.06}	{{1.39162, 1.89883, 0.}, {1.39162, 1.89883, 0.}}	{8.33861, 18.0323, 156.}	inf	746.	782.033	{359.477, 1139.65}	32.8627	0.153806	newly	2869.	5383.85	{2714., 7461.}	93.8686	0.274897	Reff	1.23601	1.33793	{1.33477, 1.34579}	8.27815	ar	40223.	49702.1	{48663., 50938.}	23.8175
